# Equimolar As_4_S_4_/Fe_3_O_4_ Nanocomposites Fabricated by Dry and Wet Mechanochemistry: Some Insights on the Magnetic–Fluorescent Functionalization of an Old Drug

**DOI:** 10.3390/ma17081726

**Published:** 2024-04-10

**Authors:** Oleh Shpotyuk, Zdenka Lukáčová Bujňáková, Peter Baláž, Andriy Kovalskiy, Małgorzata Sznajder, Jozef Cebulski, Yaroslav Shpotyuk, Pavlo Demchenko, Ihor Syvorotka

**Affiliations:** 1 Department of Optical Glass and Ceramics, O.G. Vlokh Institute of Physical Optics, 23, Dragomanov Str., 70005 Lviv, Ukraine; 2 Faculty of Mathematics and Natural Sciences, Jan Dlugosz University in Czestochowa, 13/15, al. Armii Krajowej, 42-200 Czestochowa, Poland; 3 Scientific Research Company “Electron-Carat”, 202, Stryjska Str., 79031 Lviv, Ukraine; syvorotka.jr@gmail.com; 4 Department of Mechanochemistry, Institute of Geotechnics of Slovak Academy of Sciences, 45, Watsonova Str., 04001 Košice, Slovakia; bujnakova@saske.sk (Z.L.B.); balaz@saske.sk (P.B.); 5 Department of Physics, Engineering and Astronomy, Austin Peay State University, Clarksville, TN 37044, USA; kovalskyya@apsu.edu; 6 Institute of Physics, University of Rzeszow, 1, Pigonia Str., 35-959 Rzeszow, Poland; msznajder@ur.edu.pl (M.S.); jcebulski@ur.edu.pl (J.C.); yashpotyuk@gmail.com (Y.S.); 7 Department of Sensor and Semiconductor Electronics, Ivan Franko National University of Lviv, 107, Tarnavskoho Str., 79017 Lviv, Ukraine; 8 Department of Inorganic Chemistry, Ivan Franko National University of Lviv, 6-8, Kyryla i Myfodia Str., 79005 Lviv, Ukraine; pavlo.demchenko@lnu.edu.ua

**Keywords:** arsenical–magnetite, nanomilling, X-ray powder diffraction, fluorescence

## Abstract

Multifunctional nanocomposites from an equimolar As_4_S_4_/Fe_3_O_4_ cut section have been successfully fabricated from coarse-grained bulky counterparts, employing two-step mechanochemical processing in a high-energy mill operational in dry- and wet-milling modes (in an aqueous solution of Poloxamer 407 acting as a surfactant). As was inferred from the X-ray diffraction analysis, these surfactant-free and surfactant-capped nanocomposites are *β*-As_4_S_4_-bearing nanocrystalline–amorphous substances supplemented by an iso-compositional amorphous phase (a-AsS), both principal constituents (monoclinic *β*-As_4_S_4_ and cubic Fe_3_O_4_) being core–shell structured and enriched after wet milling by contamination products (such as nanocrystalline–amorphous zirconia), suppressing their nanocrystalline behavior. The fluorescence and magnetic properties of these nanocomposites are intricate, being tuned by the sizes of the nanoparticles and their interfaces, dependent on storage after nanocomposite fabrication. A specific core–shell arrangement consisted of inner and outer shell interfaces around quantum-confined nm-sized *β*-As_4_S_4_ crystallites hosting a-AsS, and the capping agent is responsible for the blue-cyan fluorescence in as-fabricated Poloxamer capped nanocomposites peaking at ~417 nm and ~442 nm, while fluorescence quenching in one-year-aged nanocomposites is explained in terms of their destroyed core–shell architectures. The magnetic co-functionalization of these nanocomposites is defined by size-extended heterogeneous shells around homogeneous nanocrystalline Fe_3_O_4_ cores, composed by an admixture of amorphous phase (a-AsS), nanocrystalline–amorphous zirconia as products of contamination in the wet-milling mode, and surfactant.

## 1. Introduction

Multinanoparticulate (MNP) systems utilizing a few kinds of constituent nm-sized particles (nanoparticles, NPs) compose an important family of functional materials, with very attractive and important properties [1]. The MNP platform grounded on a variety of inorganic NPs, consolidated in unified nanocomposites, is one of the challenges in contemporary nanomaterials science and engineering, expanding the implementation of these substances due to a purposeful multifunctionalization [2].

Recently, this strategy was successfully utilized in the group of biomedical materials based on binary arsenic compounds referred to as arsenicals [3]. From an ancient time of outstanding achievements in traditional Chinese and Indian Ayurvedic medicines [4], these materials, represented by natural minerals such as arsenolite As_2_O_3_, realgar As_4_S_4_ and orpiment As_2_S_3_, have been in a sphere of tight interest because of their unique therapeutic efficacy in cancer treatment [5]. The main disadvantage, of bulky sulfide arsenicals (As_4_S_4_ and As_2_S_3_) possessing poor bioavailability because of low water solubility, has also been overcome due to recent progress in developed nanostructurization technologies [6]. Thus, it has been found that the transition from bulky to nanoscopic arsenicals (nanoarsenicals) could be completed by employing such nanostructurization routines as high-energy mechanical milling (MM) in dry and/or wet modes (known as nanomilling) [7,8,9], or wet solution processing chemistry [10,11,12,13]. The famous achievements on this path have been so impressive, even the emergence of a novel branch of anticancer therapy nominated as realgar nanotherapeutics has been declared recently [14]. 

In arsenical-bearing MNP systems, a nanoarsenical functionalized as the anticancer drug is often used in combination with other compounds to emerge as pharmaceuticals possessing a lot of advantages, such as fluorescence emission due to ZnS [15,16] or a magnetically addressable drug delivery ability due to Fe_3_O_4_ [17]. This approach allows functionally–compositionally inter-balanced MNP systems, like biparticulate dual-functionalized nanocomposites (e.g., anticancer-fluorescent As_4_S_4_/ZnS [18,19], or anticancer-magnetic As_4_S_4_/Fe_3_O_4_ [20,21,22]), and/or triparticulate triple-functionalized nanocomposites (e.g., anticancer-fluorescent-magnetic As_4_S_4_/ZnS/Fe_3_O_4_ [23,24]). From a purely medical point of view, what seems especially attractive is the ability to perform a few functions by one type of NP in a whole nanocomposite formulation, as expected for nanoarsenicals functionalized as quantum-confined solid fluorophores in addition to their anticancer therapeutic effect. Such a bimodal “two-in-one” functionalization foresees the surface passivation of arsenical NPs, which contribute to the non-radiative recombination from the surface states of dangling bonds (DBs), like in other nanostructured substances [25]. Noteworthily, NPs with non-passivated surfaces (stabilized in the MNP systems without surfactants) are prone to quick agglomeration and oxidation, thereby missing with time a size-limiting requirement for a quantum confinement effect (QCE) [12,24,25,26]. 

A similar argumentation concerns the magnetically addressable functionalization of magnetite Fe_3_O_4_ NPs, which is dependent on their size–domain arrangement, different in a surfactant-free (in the MNP systems fabricated by dry MM) and surfactant-capped state (in the MNP systems fabricated by a combined dry–wet processing) [27,28,29]. 

Another hallmark of MNP arsenical-bearing systems is revealed in the diversity of nanostructurization–amorphization processes governed by grinding media hosting mechanochemical transformations. Such media control both (i) the energy transfer towards the substance undergoing nanostructurization and (ii) the energy re-transfer between NPs within the nanocomposite [30]. As was shown recently [18,23,24], the nanostructurization–amorphization balance could be essentially disturbed in arsenical-bearing nanocomposites enriched in finest ZnS NPs, contributing to outer shells around greater crystallites. In these grinding media, the amorphous phase generated under continuous MM was of the same chemistry (originated from principal constituents of the nanocomposite), while the contamination effects from milling facilities could not be excluded, especially under wet-chemistry conditions. 

With this in mind, the objective of this research is to specify our understanding of the microstructure and fluorescent–magnetic functionality in biparticulate arsenical-bearing nanocomposites from an equimolar As_4_S_4_/Fe_3_O_4_ cut section fabricated by high-energy mechanochemistry in dry and combined dry–wet MM modes from initial bulky pre-cursors, respectively ensuring the surfactant-free and surfactant-capped state of both principal nanocomposite constituents (arsenical and magnetite).

## 2. Materials and Methods

### 2.1. Two-Step Mechanochemical Synthesis of Equimolar As_4_S_4_/Fe_3_O_4_ Nanocomposites

The nanocomposites fabrication route was arranged in two steps. 

In the first step, samples of a surfactant-free nanocomposite of an equimolar As_4_S_4_/Fe_3_O_4_ cut section were fabricated by dry MM using commercial arsenic monosulfide composed exclusively of a high-temperature (HT) tetra-arsenic tetrasulfide phase (*β*-As_4_S_4_) of 95% purity (Sigma-Aldrich, Saint Louis, MO, USA) and natural mineral magnetite Fe_3_O_4_ of high purity (from the mine of Kiruna, Sweden). Both bulky precursors, taken in an equimolar 1:1 ratio (5 g in total), were firstly crushed and sieved until the size of the particles approached ~200 μm, and afterwards subjected to MM for 20 min in a Pulverisette 6 (Fritsch, Idar-Oberstein, Germany) planetary ball mill in an Ar atmosphere. The MM was performed in a 250 mL tungsten carbide chamber with 50 tungsten carbide balls (394 g in total), each ball of ~10 mm in diameter, under a 500 rpm rotational speed. After MM, the powder was compressed by compacting it inside a stainless steel die under ~0.7 GPa, to produce ~1 mm disc-like pellets (~6 mm in diameter). Thereby, the pelletized samples of surfactant-free (*β*-As_4_S_4_/Fe_3_O_4_) nanocomposite were prepared via the above dry mechanochemistry route.

In the second step, dry powder from the previous step was used to prepare a nanosuspension by wet-stirred media milling in a MiniCer (Netzsch, Selb, Germany) laboratory circulation mill. Three grams of dry powdered (*β*-As_4_S_4_/Fe_3_O_4_) nanocomposite was subjected to wet MM in 300 mL of Poloxamer 407 (PX) water solution (0.5 wt. %) for 120 min, at a milling speed of 3500 rpm. Poloxamer 407 is a known nonionic surfactant composed of polyoxyethylene–polyoxypropylene triblock copolymers, which possess a good solubilizing capacity and reverse thermal gelation upon cooling, in addition to less toxicity compared with other commercially available copolymers [31,32]. The mill was loaded with 0.6 mm yttrium-stabilized ZrO_2_ balls. The nanosuspension was centrifuged at 3000× *g* rpm, the supernatant was dried at 70 °C for 2 h, and then pelletized under the same compression (~0.7 GPa). Thereby, the pelletized samples of surfactant-capped (*β*-As_4_S_4_/Fe_3_O_4_-PX) nanocomposite were prepared via a sequence of combined dry–wet powdering, conditionally referred to as wet mechanochemistry. 

The surfactant-free (*β*-As_4_S_4_/Fe_3_O_4_) nanocomposite fabricated by dry nanomilling and the surfactant-capped (*β*-As_4_S_4_/Fe_3_O_4_-PX) nanocomposite fabrication in the subsequent dry-wet nanomilling steps were examined in a few days, just after the palletization procedure (in an as-prepared state) and after a prolonged one year of natural storage in normal room temperature (RT) conditions (in an aged state).

### 2.2. Microstructure Characterization of the Pelletized Nanocomposites

The crystallographic specificity of the fabricated nanocomposites was recognized by X-ray powder diffraction (XRPD) analysis [20,21,22,23,24]. The data were collected via the transmission mode of the STOE STADI P diffractometer (STOE & Cie GmbH, Darmstadt, Germany) with a linear position-sensitive detector (the curved Ge(111) monochromator), using Cu-*K*α1 radiation. The character sizes of the crystallites of the HT tetra-arsenic tetrasulfide *β*-As_4_S_4_ phase (JCPDS No. 72-0686) [33,34] and magnetite Fe_3_O_4_ (JCPDS No. 74-0748) [35] were determined as the average apparent sizes of coherently diffracting domains derived from isotropic line broadening in the Rietveld refinement using the FullProf.2k (v.5.40) program [36].

The amorphous phase, which always appeared as an accompanying phase to synthetic *β*-As_4_S_4_ [37], was identified due to diffuse peak halos in the XRPD patterns—specifically, the first of these, the FSDP (the first sharp diffraction peak) at ~15–22°2*θ*, which is accepted as a manifestation of the medium-range structure of this arsenical over tens of Å [38,39,40]. A straightforward interpretation of this diffuse peak halo is developed within a modified microcrystalline approach [41] treating this XRPD feature as a superposition of the broadened Bragg diffraction reflections from some remnants of crystalline interplanar correlations *R*, supplemented by the Ehrenfest diffraction reflections from the most prominent pair of inter-atomic correlations belonging to these planes (*d_s_*). 

The diffuse peak halo arrangement in the XRPD patterns of the nanocomposites was parameterized using the STOE WinXPOW 3.03 and PowderCell 2.4 programs, following normalization in respect to the maximum, these data being used for the next profile fitting by the WinPLOTR program (version: July, 2011) [42,43]. The peak halo angular position (2*θ*) and full width at half maximum (FWHM) were defined with ±0.05°2*θ* accuracy, and further recalculated in the scattering vector *Q* and width Δ*Q* in a reciprocal space. The characteristic distance *R* (the spacing of the peak responsible quasi-periodicity) and correlation length *L* (serving as the operational distance for this quasi-periodicity) were determined within the known Bragg diffraction formalism. 

We also used the concept of diffuse peak halos in the XRPD patterns of amorphous substances, treating them as arising from inter-atomic distances like as in randomly packed MNP systems [44,45,46], when the collected XRPD patterns are governed by the Ehrenfest relation [46,47]:

2*d_s_*·sin*θ* = 1.23·*λ*, (1)

where *d_s_* is the average inter-atomic distance between scatterers contributing to the peak. 

### 2.3. Functional Characterization of the Nanocomposites

The magnetic functionality of the prepared nanocomposites (small ~7–8 mg pieces obtained from the pelletized samples) was tested by hysteresis loop measurements, using a VSM M-155 (PARC, Palo Alto, CA, USA) magnetometer operational in magnetic fields up to 0.3 T. The values of saturation magnetization (*M_s_*), coercivity (*H_c_*) and remanent magnetization (*M_r_*) were derived from the hysteresis curves for the as-prepared nanocomposites and for those after one year of storage in RT conditions.

The fluorescence functionality of the as-synthesized and aged nanocomposites was examined using 3D front-face fluorescence (3D-FFF) spectroscopic analysis, which is one of the efficient instrumentation tools to characterize nanostructured magnetically active substances (see, e.g., [48]). The 3D-FFF spectra (*viz*. Excitation–Emission Matrices, EEMs) were visualized employing a Horiba Fluorolog-3 spectrofluorometer, equipped with a Xenon short arc lamp used as a light source. The measurements were performed in normal RT conditions, using small pieces of pelletized nanocomposites identical to those supplied for magnetic characterization. The fluorescence emission spectra registered at 300–850 nm excitation were recorded in the 300–700 nm range using a UV/VIS R928 PMT (FL-1073) single-channel detector (Hamamatsu Photonics, Shizuoka, Japan), and reproduced as their respective spectral dependences using the integrated OriginLab software (vers. 8.5). The EEMs visualized by the contour plots of the 3D-FFF landscapes were reproduced for the nanocomposites in the as-prepared state (the measurements just after nanocomposite fabrication) and after one year of natural storage in normal RT conditions. Finally, the fluorescence emission spectra were reconstructed for the nanocomposites under excitation with several chosen wavelengths, corresponding to the maxima in the EEMs.

## 3. Results and Discussion

### 3.1. On the Microstructure Specificity of Equimolar As_4_S_4_/Fe_3_O_4_ Nanocomposites

**Figure 1:**
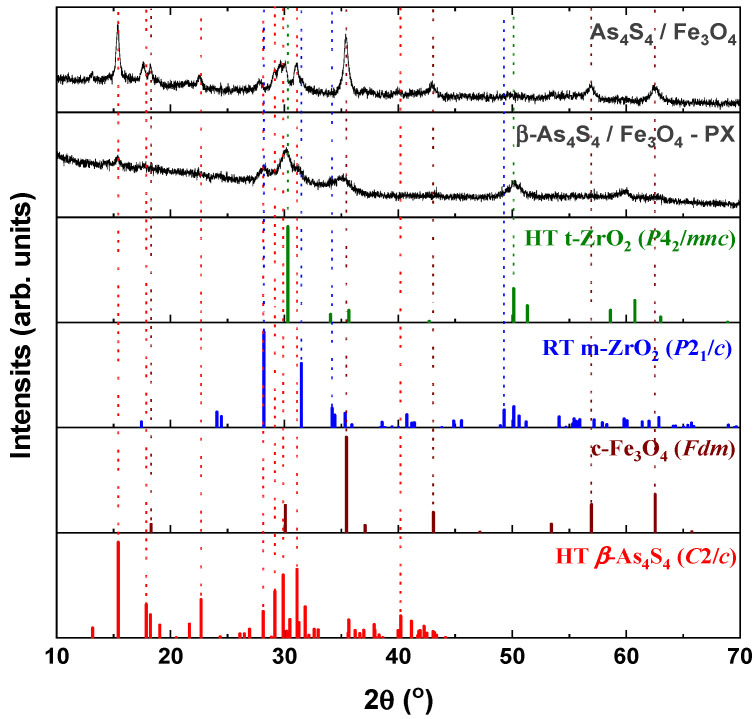
XRPD profiles of surfactant-free *β*-As_4_S_4_/Fe_3_O_4_ and surfactant-capped *β*-As_4_S_4_/Fe_3_O_4_-PX nanocomposites compared with theoretical Bragg diffraction peaks of high-temperature monoclinic *β*-As_4_S_4_ (red colored, JCPDS No. 72-0686), cubic magnetite Fe_3_O_4_ (brown colored, JCPDS No. 74-0748), room-temperature monoclinic ZrO_2_ (blue colored, JCPDS No. 37-1484) and high-temperature cubic ZrO_2_ (green colored, JCPDS No. 49-1642).

From a crystallographic point of view, both examined nanocomposites (surfactant-free *β*-As_4_S_4_/Fe_3_O_4_ and surfactant-capped *β*-As_4_S_4_/Fe_3_O_4_-PX) possess mixed nanocrystalline–amorphous structures, as follows from their XRPD patterns in Figure 1 showing the broadened Bragg diffraction peaks ascribed to the principal crystalline phases (monoclinic *β*-As_4_S_4_ [33,34] and cubic Fe_3_O_4_ [35]) against the background of suppressed but still visible diffuse peak halos positioned near ~16°2*θ*, ~32°2*θ* and ~56°2*θ*.

The tetra-arsenic tetrasulfide *β*-As_4_S_4_ phase (JCPDS No. 72-0686) [33,34] prevails in the XRPD patterns of the nanocomposites, due to strong diffraction peaks corresponding to interplanar distances *d*($\bar{1}$11) = 5.745 Å (15.41°2*θ*, *I* = 100%), *d*($\bar{2}22$) = 2.873 Å (31.11°2*θ*, I = 71.7%), *d*(221) = 2.986 Å (29.90°2*θ*, I = 65.8%), *d*(311) = 3.060 Å (29.16°2*θ*, I = 48.4%) and *d*($\bar{1}$12) = 3.917 Å (22.68°2*θ*, *I* = 40.0%). Compared to the theoretical Bragg diffraction lines ascribed to HT monoclinic *β*-As_4_S_4_ (space group, s.g. *C*2/*c*) [33,34], these peaks are broadened in width and shifted towards a lower 2*θ,* corresponding to more prolonged interplanar distances and the reduced atomic density of the nanocrystalline nc-*β*-As_4_S_4_ phase. The average crystallite sizes *D* for this phase, calculated from the broadening of the strongest line at 15.41°2*θ* (corresponding to *d*($\bar{1}$11) = 5.745 Å), approach ~19.6 nm and ~7.1 nm for surfactant-free and surfactant-capped nanocomposites. There were no detectable changes in these sizes after one year of storage of the nanocomposites in normal conditions.

The iron oxide Fe_3_O_4_ phase contributes to the XRPD patterns through broadened peaks ascribed to interplanar distances in the cubic structure (s.g. *Fd*$\bar{3}$*m*) of mineral magnetite (JCPDS No. 74-0748) [35]), the strongest ones being as follows: *d*(220) = 2.968 Å (30.09°2*θ*, I = 28.5%), *d*(311) = 2.531 Å (35.44°2*θ*, *I* = 100%), *d*(400) = 2.099 Å (43.07°2*θ*, I = 21.2%), *d*(333) = 1.615 Å (56.96°2*θ*, I = 29.5%) and *d*(440) = 1.484 Å (62.55°2*θ*, I = 40.0%). Compared to the theoretical lines of the magnetite Fe_3_O_4_ phase, there are no visible changes in the angular positions of these peaks, which are not reproducible in a diffuse shape character for amorphous substances (Figure 1), testifying that the MM-driven nanostructurization of this phase is not accompanied by an amorphization of the parent crystalline phase. The average crystallite sizes *D* for nanocrystalline nc-c-Fe_3_O_4_, estimated from the broadening of the strongest line at 35.44°2*θ* (ascribed to *d*(311) = 2.531 Å), approach ~12.8 nm and ~5.0 nm for the surfactant-free and surfactant-capped nanocomposites, respectively. Nevertheless, the last value derived for the *β*-As_4_S_4_/Fe_3_O_4_-PX nanocomposite should be accepted with some precautions, in view of the contamination from zirconia milling balls activated under wet processing. 

**Figure 2:**
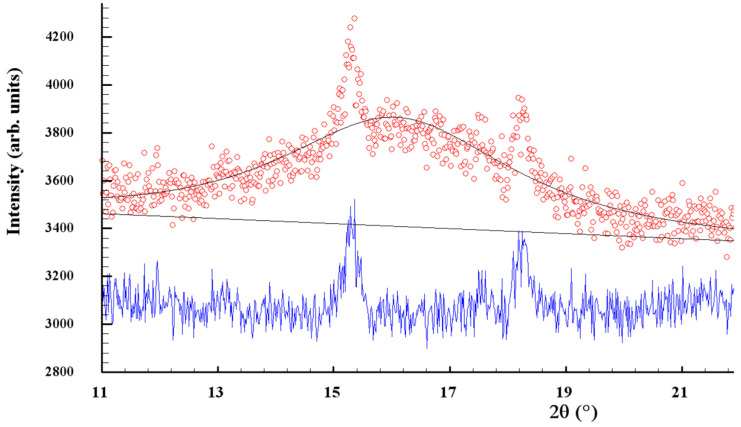
Experimental (red points) and calculated (black solid line) XRPD pattern of surfactant-free *β*-As_4_S_4_/Fe_3_O_4_ nanocomposite, showing the profile fitting of the FSDP overlapped with broadened peaks of nanocrystalline nc-*β*-As_4_S_4_ at 2*θ* angles of 15.41°, 17.87° and 18.24° (the difference curve is given by the blue solid line at the bottom).

The clearly decaying background and broad peak halos on the XRPD profiles, near diffraction angles 2*θ* approaching ~16°, 32° and 56° (see Figure 1), speak in favor of an additional amorphous phase stabilized in the nanocomposites. As was proved previously [49,50], this phase is continuously generated in arsenic sulfides under high-energy MM from different sources, specifically, the macroscopic crystalline *β*-As_4_S_4_ and amorphous phase initially present among bulky precursors. Whichever the case, this amorphous phase (a-AsS), either appearing under nanostructurization from a bulky crystalline counterpart (as a newly amorphized phase) or derived from an initial amorphous phase (as a re-amorphized phase), is iso-compositional to the precursor crystalline phase of arsenic monosulfide a-AsS (compositionally equivalent to tetra-arsenic tetrasulfide, As_4_S_4_). The detailed inspection of the XRPD pattern for surfactant-free *β*-As_4_S_4_/Fe_3_O_4_ nanocomposite in the 2*θ* region, covering the FSDP-related peak halo after subtracting the overlapping peaks of nc-*β*-As_4_S_4_ at 15.41°2*θ*, 17.87°2*θ* and 18.24°2*θ* (Figure 2), allows a refinement of the FSDP parameters ascribed to this a-AsS phase. In Table 1, these parameters are compared with those ascribed to the compositionally authentic amorphous phase derived by dry MM from other *β*-As_4_S_4_-bearing MNP composites (mono-, bi- and even triparticulate) [20,23,49,50]. Unfortunately, it was not possible to reliably parametrize the derived amorphous phase in the surfactant-capped *β*-As_4_S_4_/Fe_3_O_4_-PX nanocomposite, since the FSDP-related diffuse peak halo in this case was slightly revealed at the growing background of the collected XRPD pattern (see Figure 1).

It is worth mentioning that, in the XRPD patterns of *β*-As_4_S_4_/Fe_3_O_4_ nanocomposite (see Figure 1 and Figure 2), the FSDP-related peak halo is well expressed, as compared with the peak halo in the bulky precursor (that is, unmilled *β*-As_4_S_4_) [49,50], being somewhat enhanced inwidth (to Δ*Q* = 0.33 Å^−1^, see Table 1) and shifted towards lower diffraction angles 2*θ,* corresponding to a reduced scattering vector *Q* = 1.140 Å^−1^. This growing amorphization trend is well pronounced with an increased fraction of Fe_3_O_4_ NPs, observed in Table 1 in the row of growing particularity of *β*-As_4_S_4_-bearing nanocomposites (in transition from mono-particulate *β*-As_4_S_4_ to biparticulate nanocomposites such as 4·*β*-As_4_S_4_/1·Fe_3_O_4_ and *β*-As_4_S_4_/Fe_3_O_4_, and to triparticulate *β*-As_4_S_4_/4·ZnS/Fe_3_O_4_ nanocomposite) [50]. Such an abundant solid-state amorphization in the examined *β*-As_4_S_4_-bearing nanocomposites, obeying a “shell” kinetic model, is probably caused by an enhanced effect in the mill due to great number of hard NPs acting as additional grinding agents.

Within a modified microcrystalline approach [37], the FSDP ascribed to the compositionally authentic amorphous phase derived under dry MM can be treated as originating from the averaged contribution of the interplanar correlations belonging to the crystalline remnants of the HT *β*-As_4_S_4_ phase (with quasi-periodicity *R* = 5.51 Å and correlation length *L* = 24.7 Å), superimposed by prominent inter-atomic correlations belonging to these remnants with the *d_s_* distance approaching 6.78 Å (Table 1). Assuming atomic packing in the amorphized substance, as derived from the superposition of layer and molecular arsenical structures, the density of the newly derived a-AsS phase is expected to be ~3.43 g·cm^−3^ [23]. Thereby, the MM-driven amorphization in the surfactant-free *β*-As_4_S_4_/Fe_3_O_4_ nanocomposite can be imagined as a transition from monoclinic *β*-As_4_S_4_ built of cage-like As_4_S_4_ molecules [34] to a disordered network built of some derivatives from these molecules, the most plausible configurations being dependent on the energy transferred to bulk arsenical undergoing nanostructurization [50]. Thus, a strict size identification of the nanocrystalline phases after wet MM in poloxamer aqueous solution is problematic, because of the enormous increase in the amorphization of the principal arsenical phase. This effect is enhanced by contamination from yttrium-stabilized zirconium oxide ZrO_2_ milling balls, forming fine (~10–15 nm sized [51]) crystallites acting as additional extra-hard grinding media. The final products of contamination are recognized as different zirconia polymorphs, in part: RT monoclinic m-ZrO_2_ (s.g. *P*2_1_/*c*) stable below 1172 °C, HT tetragonal t-ZrO_2_ (s.g. *P*4_2_/*mnc*) stable at 1172–2347 °C, HT cubic c-ZrO_2_ (s.g. *Fm*$\bar{3}$*m*) stable above 2347 °C and amorphous a-ZrO_2_ [51,52,53,54]. 

The XRPD pattern of the *β*-As_4_S_4_/Fe_3_O_4_-PX nanocomposite fabricated by wet MM (see Figure 1) confirms the formation of a mixed tetragonal–monoclinic phase, with a possible admixture of a-ZrO_2_, like in nanocrystalline nc-ZrO_2_ obtained by MM [51] or precipitation from m-ZrO_2_ aqueous solutions [52]. Within the (25–40)°2*θ* domain, the HT modification of t-ZrO_2_ (matched with JCPDS No. 81–1455 [52]) is revealed due to diffraction peaks at 2*θ* approaching 30.27° and 35.22°, which correspond to reflections from the (101) and (110) lattice planes, whereas the RT zirconia m-ZrO_2_ (matched with JCPDS No. 37–1484 [53]) is revealed due to peaks positioned at 28.33° and 31.54° corresponding to the ($\bar{1}$11) and (111) plane of the monoclinic phase (see Figure 1). These peaks are broadened due to the nanostructurization of both polymorphs [51,52] and superimposed with pronounced peaks of nc-*β*-As_4_S_4_, positioned at 29.16°2*θ* (48.4%), 29.90°2*θ* (65.8%) and 31.11°2*θ* (71.7%), so that the strongest peak in the XRPD pattern of c-Fe_3_O_4_ at 35.44°2*θ* is overlapped with a reflection from the (110) plane in nc-t-ZrO_2_ at 35.22°2*θ* (see Figure 1). So, the determination of the crystallite sizes of magnetite derived by wet MM is problematic in this angular domain; the above value is accepted only as a very rough estimation. Under zirconia contamination, the gradual decrease in the diffraction peaks ascribed to both the arsenical and magnetite principal phases is obvious (Figure 1), just as this occurs in polyethylene glycol-coated nc-Fe_3_O_4_ [55]. The preponderance of contamination products, in the form of nanocrystalline–amorphous zirconia polymorphs, in the examined nanocomposites fabricated by wet MM indicates that the crystalline behavior of both constituents (monoclinic nc-*β*-As_4_S_4_ and cubic nc-c-Fe_3_O_4_) is highly suppressed. 

### 3.2. On the Fluorescence Functionalization of As_4_S_4_/Fe_3_O_4_ Nanocomposites

**Figure 3:**
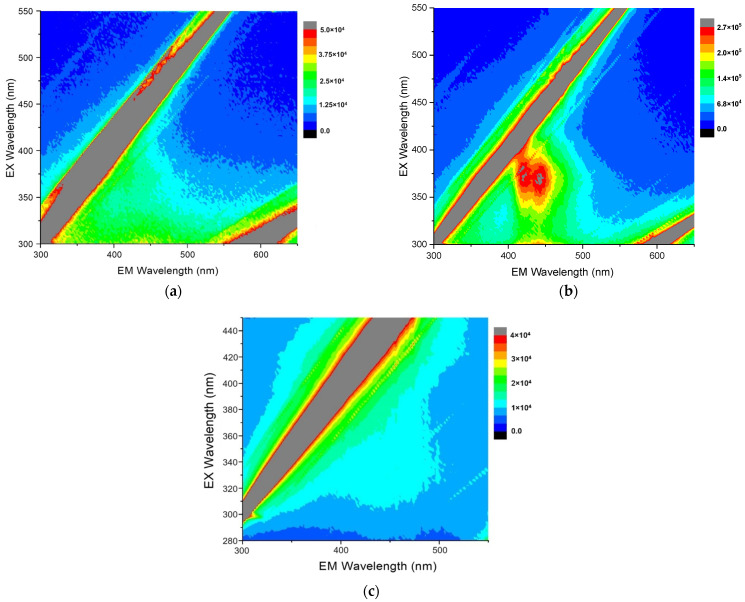
Contour plots of the 3D-FFF landscapes reproduced for surfactant-free *β*-As_4_S_4_/Fe_3_O_4_ (**a**) and surfactant-capped *β*-As_4_S_4_/Fe_3_O_4_-PX nanocomposites (**b**,**c**) in the as-prepared state (**a**,**b**) and after one of year storage in normal RT conditions (**c**). The diagonal gray-colored straight stripes in the EEM correspond to first- and second-order Rayleigh scattering of the excitation light.

The fluorescence and magnetic functionalities of the arsenical-bearing MNP systems like the equimolar As_4_S_4_/Fe_3_O_4_ nanocomposites are intricate, because they are tuned to a great extent not only by the NPs themselves (their geometrical sizes and shapes) but also by their interfaces, which are essentially dependent on the time during natural storage after nanocomposite fabrication [10,11,12,28]. Typical fluorescent landscapes, showing the respective EEMs reproduced from the 3D-FFF data for equimolar As_4_S_4_/Fe_3_O_4_ nanocomposites in the as-prepared state and after one year of storage in normal RT conditions, are shown in Figure 3. 

It is clear there is no fluorescence emission in the *β*-As_4_S_4_/Fe_3_O_4_ nanocomposite fabricated by dry powdering (without any surfactants), apart from gray-colored diagonal stripes corresponding to the first-order (in the bottom-right corner) and second-order (in the top-left corner) Rayleigh scattering of the excitation light (see Figure 3a). In contrast, a prominent fluorescence emission, revealed in two peaks at ~410–420 nm and ~440–450 nm in response to excitation with ~360–390 nm wavelengths, is revealed in the 3D-FFF landscape for the as-prepared *β*-As_4_S_4_/Fe_3_O_4_-PX nanocomposite fabricated by powdering in the dry–wet mode (see Figure 3b). However, this effect is unstable, decaying with time, so there was no emission in this sample after one year of storage in normal RT conditions (see Figure 3c). 

**Figure 4:**
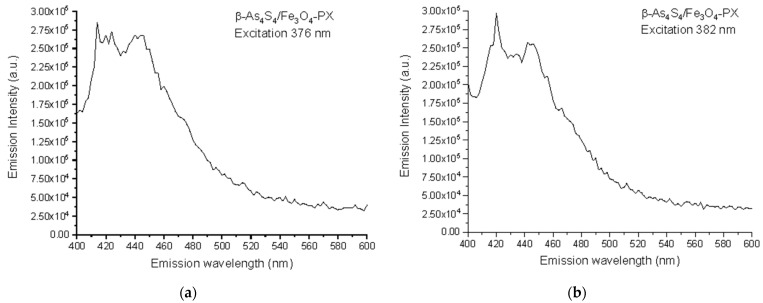
Spectral dependences of fluorescence emission in as-prepared specimens of surfactant-capped *β*-As_4_S_4_/Fe_3_O_4_-PX nanocomposite under excitation at 376 nm (**a**) and 382 nm (**b**).

Based on the collected 3D-FFF data, the fluorescence emission spectra, under a light excitation of two wavelengths of 376 nm (3.30 eV) and 382 nm (3.24 eV), were reconstructed for the surfactant-capped *β*-As_4_S_4_/Fe_3_O_4_-PX nanocomposite in the as-prepared state, these spectra being reproduced in Figure 4a,b, respectively. The broad fluorescence emission in the 400–500 nm range is composed of two strong peaks at ~412–422 nm (corresponding to 417 nm in average, *viz*. 2.97 eV) and ~438–445 nm (442 nm in average, *viz*. 2.80 eV), and three shoulders, better distinguishable under excitation with a longer wavelength of 382 nm (strong shoulder near ~455 nm, weak shoulder near ~470 nm and very weak shoulder near ~485 nm, respectively corresponding to 2.72 eV, 2.64 eV and 2.55 eV).

The similar shaping and positioning of the fluorescence emission bands were also observed in colloidal solutions of arsenic(II) sulfide quantum dots (QDs) by Junzhong Wang et al. [10]. It was found that nanocrystals of arsenic(II) sulfide (which is a crystallographic prototype of HT *β*-As_4_S_4_, but not RT α-As_4_S_4_ nominated as a realgar [34]) display the features associated with typical QCE, such as a blue shift in absorption and near-band-edge emission. Specifically, the 7–10 nm quantum dots (QDs) possess a triple-peak excitation at 355 nm (*viz*. 3.49 eV), 373 nm (3.32 eV) and 395 nm (3.14 eV), and a near-band-gap blue-cyan emission owing to strong peaks at 408 nm (*viz*. 3.04 eV) and 433 nm (2.86 eV), a shoulder at 455 nm (2.72 eV) and a weak band near ∼485 nm (2.55 eV). With an increase in the excitation wavelength, a negligible shift was observed in the fluorescence emission, suggesting that multiple peaks are not simply due to the size heterogeneity of these QDs, but rather due to the presence of near-band-gap and defect-related shallow donor and acceptor or/and deep-trapping states, like in wide-bandgap nanocrystals [56,57]. With a further decrease in the size of the QDs (to 4–3 nm), both the excitation and emission maxima shift towards the shorter wavelength (corresponding to higher photon energies *hν*). 

Let us recognize the origin of the fluorescence emission in the *β*-As_4_S_4_/Fe_3_O_4_-PX nanocomposite (see Figure 4), using an analogy between nc-*β*-As_4_S_4_ and wide-bandgap nanocrystals such as ZnS [56,57]. The bulk As_4_S_4_ has an energy gap for the intrinsic generation of holes and electrons (the bandgap energy *E_g_*) close to 2.3–2.4 eV [58,59]. Hence, under over-bandgap excitation with photon energies *hν* > *E_g_* (e.g., 2.62 eV as in [60]), the fluorescence spectra of this specimen dominate with a broad near-bandgap emission in a visible range (e.g., around 563 nm or 2.2 eV as in [60]). Because of the QCE, the maximum of optical absorption in nc-*β*-As_4_S_4_ is shifted towards shorter wavelengths below ~360 nm (~3.44 eV), corresponding to the *E_g_* more than in the bulk counterpart. The multipeak fluorescence excitation may simply result from multimodal NPs distribution, as was observed in the case of 7–10 nm sized NPs [10]. In respect to the fluorescence emission spectra reconstructed in Figure 4, we suppose (in analogy to colloidal ZnS NPs studied in [57]) that defects of the crystalline lattice, such as interstitial and vacant sites which appeared as a result of deformations and strains due to mechanical attrition, are responsible for the multiband blue-cyan fluorescence emission in nc-*β*-As_4_S_4_. Since the extent of intrinsic deformation depends on the size of an interstitial atom and the covalent radius of As (~120 pm) is higher than that of S (~103 pm) [61], the interstitial As induces more strain into the crystalline network, with the result that electrons originating from this interstitial As atom have smaller binding energies, and their energy levels should be localized closer to the conduction band edge than those of the interstitial S atom to the valence band edge.

Therefore, we attribute the 417 nm emission peak (*viz*. 2.97 eV) in Figure 4 to the interstitial As atom andhe t442 nm peak (*viz*. 2.80 eV) to the interstitial S atom in the lattice of nc-*β*-As_4_S_4_. The strain depends also on the specific vacant space ascribed to the atom, so that electron levels of larger As atoms (As vacancies) are stretched deeper into the bandgap than S vacancies. Therefore, we attribute the strong shoulder at 455 nm (*viz*. 2.72 eV) in the emission spectrum in Figure 4 to As vacancies, and the weak 470 nm (*viz*. 2.64 eV) shoulder to S vacancies. Finally, the slightly distinguishable fluorescence emission revealed in the very weak 485 nm shoulder (Figure 4) can be ascribed to surface defect states, preferentially, the DB of S atoms for chalcogenides [61].

**Figure 5:**
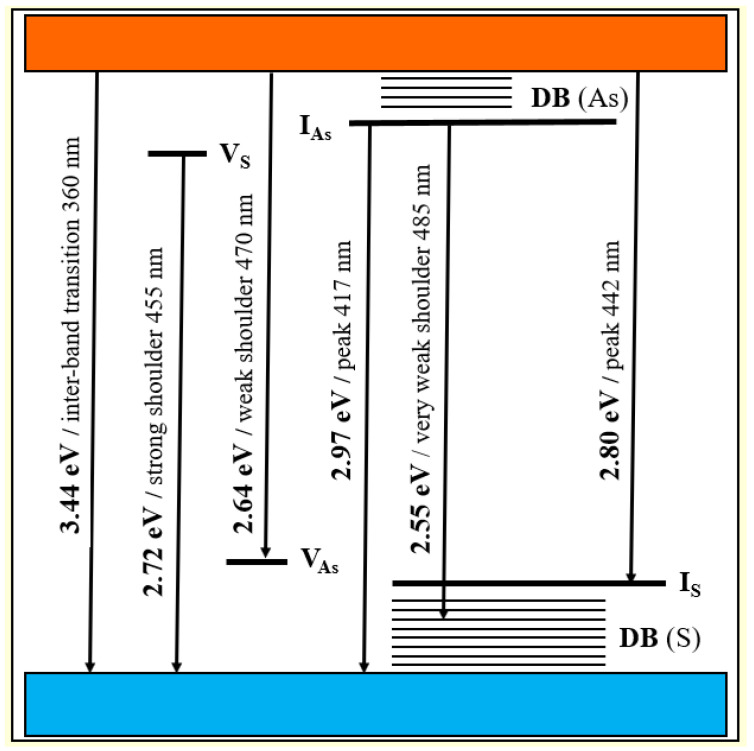
Schematic energy level diagram for defect-related intrinsic and surface states in nc-β-As_4_S_4_.

The energy diagram for defect-related intrinsic and surface states in nc-*β*-As_4_S_4_ is shown in Figure 5. The origin of defect-related states in nc-*β*-As_4_S_4_ can be reinterpreted in terms of a coordination of topological defects model proper to chalcogenide glass networks [61]. Within this model, the donor level of the S vacancy near the bottom of conduction band (V_S_) can be accepted as the S_1_^−^ defect (the sub-script and super-script are respectively used to denote the coordination and charge state of the atom), while the acceptor level of the As vacancy near the top of valence band (V_As_) can be accepted as the As_4_^+^ defect. Similarly, the donor level of the interstitial As atom in the upper part of the bandgap (I_As_) is operational rather as the As_2_^−^ defect, whereas the acceptor level of the interstitial S atom (I_S_) in the bottom of the bandgap behaves like the S_3_^+^ defect.

The fluorescence properties of the NPs possessing a QCE are known to be strongly dependent on the interfacial processes at the boundaries between the nanocrystallites and the surrounding medium [25,26]. The complete disappearance of fluorescence in the surfactant-capped *β*-As_4_S_4_/Fe_3_O_4_-PX nanocomposite after one year of natural storage testifies in favor of a substantial degradation of such interface states ascribed to arsenical nanocrystallites possessing CFE. This may occur due to (i) an increased non-radiative recombination channel from the restored defect-related surface states inhibited in the as-prepared composite by the surfactant, and/or (ii) the agglomeration of arsenical nanocrystallites because of the destroyed surfactant layer. XRPD analysis testifies that we rather deal with the former, since the sizes of the arsenical nanocrystallites did not change considerably under storage. In fact, the nc-*β*-As_4_S_4_ species contributing to the fluorescence through CFE compose a specific homogeneous core covered by a heterogeneous shell consisting of a-AsS [62,63] and the capping agent (Poloxamer 407). Therefore, the as-prepared nanocrystallites are reliably protected against agglomeration, while defect states at their interfaces can be affected by some changes under growing physical ageing.

Noteworthily, a partial destruction of the core–shell architecture of the NPs, without dramatic changes in the average hydrodynamic diameter of the coalescing nc-*β*-As_4_S_4_ species (~100–150-nm), was observed in two-years stored As_4_S_4_/ZnS/Fe_3_O_4_ nanocomposites capped by Poloxamer 407 and folic acid [24]. It could be assumed that such extended NPs possess heterogeneous shell structures, where the inner shell interfaces, composed preferentially of amorphous phase (a-AsS), are more loosely packed in comparison to the homogeneous nanocrystalline cores and therefore enriched in different structural defects contributing to fluorescence quenching by enhanced non-radiative recombination, and the outer shell interfaces, additionally hosting the capping agent, are reduced in such defects (resulting in an enhanced emission due to an inhibited non-radiative recombination channel). The destruction of such a heterogeneous core–shell architecture occurs mainly in the outer shell, so that it can be realized without any dramatic changes in the crystallites forming the cores. That is why the increased thickness of the inner shell (enriched in a-AsS), acting as a non-radiative site for the recombination of carriers in biparticulate *β*-As_4_S_4_/Fe_3_O_4_-PX composites, in comparison with the triparticulate As_4_S_4_/ZnS/Fe_3_O_4_-PX ones [24], results in the higher instability of the former.

A better stability of MNP systems can be also achieved if one of the constituents forms the shell, around other nanocrystallites forming the core. This is often achieved in arsenical-bearing nanocomposites using zinc sulfide ZnS NPs with sizes one order less than the sizes of the other constituents, like in biparticulate Poloxamer-capped As_4_S_4_/ZnS nanocomposites [18,19].

### 3.3. On the Magnetic Co-Functionalization of Fluorescent As_4_S_4_/Fe_3_O_4_ Nanocomposites

As to the magnetically addressable functionality of fluorescent equimolar As_4_S_4_/Fe_3_O_4_ nanocomposites, the intrinsic architectures of cubic magnetite nanocrystallites nc-c-Fe_3_O_4_ are the most crucial [21].

The RT hysteresis loop of the *β*-As_4_S_4_/Fe_3_O_4_ nanocomposite fabricated by nanomilling in a dry mode testifies in favor of its ferromagnetic origin, giving a saturation magnetization *M_s_*, coercivity *H_c_* and remanent magnetization *M_r_* respectively approaching 26.48 A·m^2^/kg, 3.3 kA/m and 5.98 A·m^2^/kg [22].

The RT magnetization in the bulk inverse-spinel-structure magnetite Fe_3_O_4_ is known to achieve the maximal value, approaching *M_s_^bulk^*~92 A·m^2^/kg [64,65,66]. The reduced saturation magnetization *M_s_* in this counterpart fabricated by nanostructurization without any surfactants is known to be achieved due to the finite size effect [65,66,67,68,69]. Thus, the *M_s_* value of 70 nm sized Fe_3_O_4_ NPs prepared by the hydrothermal method was 68.7 A·m^2^/kg [68], while the 10 nm sized NPs prepared via sonochemical synthesis have an *M_s_* < 1.25 A·m^2^/kg [69]. Because of a negligible coercive force, the emerging of RT supermagnetism was declared in these NPs [68,69], due to crystallites with character sizes below the critical size of single Fe_3_O_4_ domains (~54 nm) [70]. Noteworthily, the saturation magnetization of ~8 nm sized Fe_3_O_4_ NPs obtained by MM in conditions similar to actual in this research was *M_s_* = 69.2 A·m^2^/kg, while in the biparticulate Fe_3_O_4_/4·ZnS nanocomposite with average sizes of Fe_3_O_4_ crystallites ~22 nm, the *M_s_* value was reduced to 34.5 A·m^2^/kg [66]. Of course, the latter was also achieved due to the minor content of the ferromagnetic component in the nanocomposite prepared by dry MM, as can be expected from the reduced *M_s_* = 14.7 A·m^2^/kg observed in the triparticulate *β*-As_4_S_4_/4·ZnS/Fe_3_O_4_ nanocomposite with smaller fraction of magnetite Fe_3_O_4_ [22].

A further decrease in the saturation magnetization *M_s_* is achieved in MNP systems based on core–shell structured magnetite NPs, owing to surface spin disordering [27,71]. The magnetically dead surface layer around these crystallites (nc-Fe_3_O_4_) does not originate from the same Fe_3_O_4_ component (clearly showing a cubic crystalline nature [20,21,22,23,24]) or the surfactant (which should be too extended to ensure a measurable decrease in the *M_s_* value). Thus, the authors [28] assumed that the magnetic co-functionalization of fluorescent-Fe_3_O_4_-ZnS nanocomposites was adjusted by the core–shell architecture of the constituent NPs (controlling the ratio of the finest ZnS crystallites forming the shells around more extended cores of magnetite crystallites). In MM-derived As_4_S_4_/Fe_3_O_4_ nanocomposites, this is performed by an amorphized substance originating from the other principal constituents, like amorphous arsenic monosulfide (a-AsS) generated from the arsenical phase (*β*-As_4_S_4_). This effect could be enhanced by the polymeric nature of the surfactant, as it was in surface-coated sodium bis(2-ethylhexyl)sulfosuccinate used for the hydrothermal synthesis of ~27 nm sized Fe_3_O_4_ crystallites possessing an *M_s_* = ~3–4 A·m^2^/kg [67].

Thus, it seems reasonable that the lower magnetization in the surfactant-free *β*-As_4_S_4_/Fe_3_O_4_ nanocomposite (*M_s_* = 26.48 A·m^2^/kg [22]), as compared with the bulk counterpart (with *M_s_^bulk^*~92 A·m^2^/kg [64]), is most likely attributed to the finite size effect of ~12.8 nm Fe_3_O_4_ crystallites (forming magnetically active cores), enhanced by a magnetically dead shell composed preferentially of an amorphous substance generated under dry MM (such as a-AsS), creating spin disordering on the surfaces of the magnetite crystallites. Assuming each magnetic NP as consisting of such a nanocrystalline core (nc-Fe_3_O_4_) having a magnetic structure character similar to bulk magnetite and a magnetically disordered shell, as suggested by Chen et al. [71], the thickness of the magnetically inert layer in the *β*-As_4_S_4_/Fe_3_O_4_ nanocomposite is estimated to be ~1–1.2 nm, reflecting the complexity in a possible core–shell arrangement of the magnetic NPs. The low coercivity in this nanocomposite (*H_c_* = 3.3 kA/m) testifies in favor of an increased volume of nc-Fe_3_O_4_ cores under the unchanged magnetic anisotropy of the NPs. The reduced remanence or squareness *SQ,* defined by the *M_r_*/*M_s_* ratio in this surfactant-free nanocomposite, is only ~0.23, suggesting that magnetically addressable NPs fabricated by dry MM are rather single-domain entities possessing a uniaxial anisotropy. 

**Figure 6:**
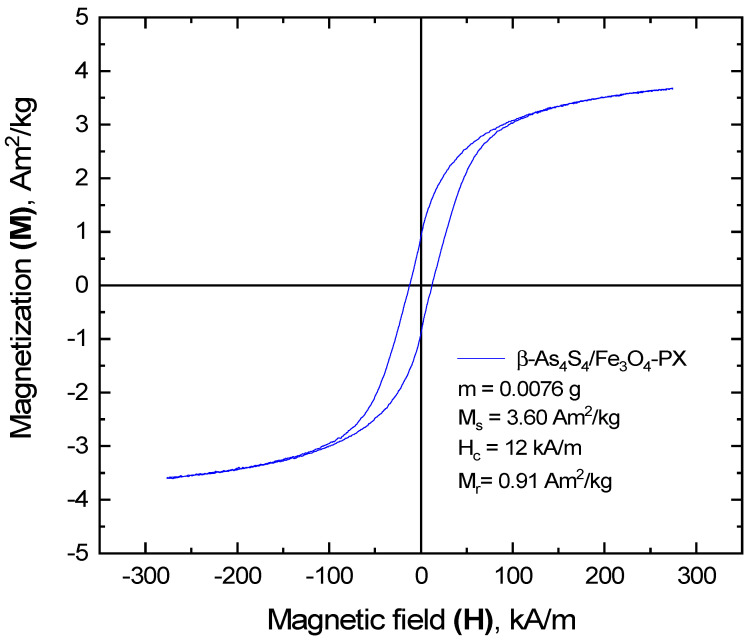
Ferromagnetic hysteresis loop of surfactant-capped *β*-As_4_S_4_/Fe_3_O_4_-PX nanocomposite after one year of storage in normal RT conditions.

The more considerable changes in magnetic functionality occur in the surfactant-capped *β*-As_4_S_4_/Fe_3_O_4_-PX nanocomposite fabricated by wet MM in the second step. As inferred from Figure 6, the hysteresis loop of this nanocomposite becomes more depressed in comparison with the dry-nanomilled sample, while being further well pronounced in ferromagnetic behavior, resulting in significantly modified values of saturation magnetization *M_s_*≈3.6 A·m^2^/kg, coercivity *H_c_*≈12 kA/m and remanent magnetization *M_r_*≈0.9 A·m^2^/kg. In fact, the invariant squareness *SQ*, approaching ~0.25 in this sample, accompanied by a reduced *M_s_* and *H_c_*, is consistent with the unchanged magnetic-domain structure of the magnetically active cores under more expanded magnetically inert shells. 

Following the linear variation of the saturation magnetization *M_s_* with the reciprocal of the average diameter of the NPs [27,71], the effective thickness of this magnetically inert shell is estimated to be ~1.5–1.6 nm. Since the increase in the coercivity *H_c_* with the reduced sizes of the NPs is consistent with an increase in the anisotropy constant *K* and a decrease in the *M_s_* [27], a reduced anisotropy is expected in magnetic NPs in the surfactant-capped nanocomposite, which can be associated with an expansion of heterogeneous shells around almost unchanged homogeneous cores. At the basis of the XRPD analysis, these heterogeneous magnetically inert shells around homogeneous Fe_3_O_4_-bearing cores (nc-Fe_3_O_4_) possess a magnetic disordering character from the admixture of a few substances, such as (i) a-AsS originating from the arsenical phase (*β*-As_4_S_4_) undergoing nanostructurization, (ii) nanocrystalline–amorphous zirconia as products of contamination from wet-MM and (iii) Poloxamer 407 as a nonionic surfactant from the group of polyoxyethylene–polyoxypropylene triblock copolymers, acting as capping agent.

### 3.4. Specific Core–Shell Arrangement of Crystallites Tuning Magnetic–Fluorescent Functionalization of As_4_S_4_/Fe_3_O_4_ Nanocomposites

**Figure 7:**
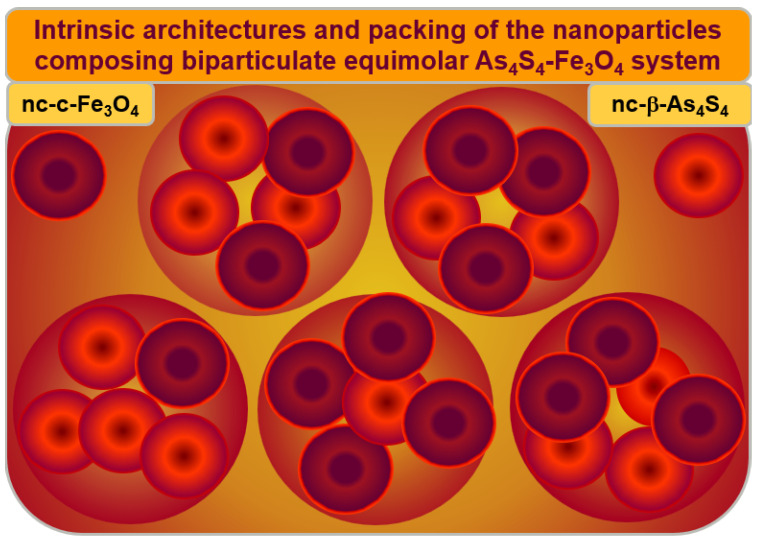
Hierarchical packing of the principal constituents composing As_4_S_4_-Fe_3_O_4_ nanocomposite. The arsenical–magnetite nanocrystallites in an almost 1:1 ratio (denoted by large circles) are stabilized in a specific core–shell arrangement of individual nanocrystallites (nc-*β*-As_4_S_4_ and nc-c-Fe_3_O_4_), possessing intrinsic architectures (distinguished by small circles), where more or less homogeneous nanocrystalline cores (dark brown colored) are surrounded by size-extended heterogeneous shells (bright red colored), preferentially of a mixed nanocrystalline–amorphous nature.

The hierarchical packing of constituent NPs in the Poloxamer-capped *β*-As_4_S_4_/Fe_3_O_4_-PX nanocomposite is illustrated in Figure 7. Both principal constituents of these biparticulate nanocomposites (monoclinic nc-*β*-As_4_S_4_ and cubic nc-c-Fe_3_O_4_ nanocrystallites in an almost equimolar 1:1 ratio) form a specific heterogeneous core–shell arrangement, splitting the intrinsic architectures of these constituents, based on more or less homogeneous nanocrystalline cores and strongly heterogeneous nanocrystalline–amorphous shells. 

The nc-*β*-As_4_S_4_ cores (dark red colored in Figure 7) are surrounded by an amorphous a-AsS substance forming the inner interface of the shell (bright red colored on Figure 7), enriched in DB and/or other structural defects acting as effective channels of the non-radiative recombination of carriers, while the outer shell interface is more saturated by the capping agent (Poloxamer 407). The blue-cyan fluorescence emission prevails when nc-*β*-As_4_S_4_ cores obeying the QCE are stabilized in the nearest environment of the surface-passivated capping agent (as in the as-prepared *β*-As_4_S_4_/Fe_3_O_4_-PX nanocomposites fabricated by wet MM in Poloxamer 407 aqueous solution), while amorphous phase (a-AsS) without any capping agents merely quenches the fluorescence (as in the surfactant-free *β*-As_4_S_4_/Fe_3_O_4_ nanocomposite prepared by dry MM). Only *β*-As_4_S_4_ nanocrystallites forming homogeneous cores passivated by surfactant contribute to fluorescence emission, while heterogeneous shells composed of the amorphized arsenical (and other products of possible contamination from wet-processing routes, like nanocrystalline–amorphous zirconia) lead to fluorescence quenching. The non-radiative recombination inhibited by the surfactant in the as-fabricated state of these nanocomposites can be restored after one year of natural storage of the samples, resulting in a complete (or partial) disappearance of fluorescence. 

Similarly, the magnetite nc-c-Fe_3_O_4_ nanocrystallites form homogeneous magnetically active cores (dark red colored in Figure 7) in a heterogeneous environment of magnetically inert size-extended shells, presumably composed by the admixture of a few substances such as amorphous phase (a-AsS), originating from nanostructurized arsenical (nc-*β*-As_4_S_4_); nanocrystalline–amorphous zirconia, appearing as a product of contamination in the dry-MM mode; and a nonionic surfactant such as Poloxamer 407. 

Thus, the extended heterogeneous shell surrounding a more or less homogeneous core composed of nc-c-Fe_3_O_4_ plays a crucial role in the magnetic co-functionalization of the *β*-As_4_S_4_/Fe_3_O_4_-PX nanocomposite. The magnetically active, structurally homogeneous nc-c-Fe_3_O_4_ core appears to be reliably protected against agglomeration owing to the magnetically inert shell, which can be further expanded in size during amorphization in the nanocomposite undergoing a prolonged ageing (storage in normal RT conditions), resulting in decreased magnetization in a whole MNP system. 

## 4. Conclusions

The hallmarks determining the fluorescence–magnetic co-functionalization of biparticulate equimolar As_4_S_4_/Fe_3_O_4_ nanocomposites derived from their coarse-grained constituents (monoclinic tetra-arsenic tetrasulfide *β*-As_4_S_4_ and cubic magnetite c-Fe_3_O_4_) by two-step mechanochemical processing in a high-energy ball mill, operational in dry- and dry–wet-nanomilling modes, are as follows:

1. The arsenical component is typically stabilized in the nm-sized high-temperature monoclinic nc-*β*-As_4_S_4_ phase, supplemented by a great amount of a continuously generated iso-compositional amorphous a-AsS substance, testifying in favor of the “shell” kinetic model of nanomilling-driven solid-state amorphization.
2. The surfactant-capped nanocomposites fabricated in an aqueous Poloxamer solution can be subjected to contamination from the applied wet-milling facilities. Nanocrystalline–amorphous zirconia, as main products of such contamination, can essentially suppress the crystalline behavior of the principal nanocomposite constituents.
3. The fluorescence and magnetic properties of the nanocomposites derived by dry or wet mechanochemistry are intricate, being tuned not only by the sizes of the constituent nanoparticles, but also by their interfaces, dependent on natural ageing after nanocomposite sample fabrication.
4. The surfactant-stabilized arsenical crystallites (nc-*β*-As_4_S_4_) obeying nm-sized quantum confinement, can be stabilized as efficient fluorescent media possessing multicomponent emission in the blue region of the spectrum. A specific core–shell arrangement, consisting of inner and outer shell interfaces hosting an amorphous a-AsS phase and capping agent, is responsible for the blue-cyan fluorescence peaking at ~417 nm and ~442 nm in the as-fabricated surfactant-capped *β*-As_4_S_4_/Fe_3_O_4_-PX nanocomposites. The disappearance of this fluorescence under the long-term natural storage of the nanocomposites is explained in terms of the destroyed core–shell architectures of the constituent nanoparticles.
5. The magnetic functionalization of both the surfactant-free and surfactant-capped As_4_S_4_/Fe_3_O_4_ nanocomposites is determined by their size-extended heterogeneous shells (around homogeneous nc-c-Fe_3_O_4_ cores), which possess a structural disordering character from the admixture of an amorphous a-AsS phase derived from nanostructured arsenical, agents of possible contamination (such as nanocrystalline–amorphous zirconia) and the surfactant (such as Poloxamer 407).

## Figures and Tables

**Table 1:** The FSDP parameterization in β-As_4_S_4_-bearing nanocomposites fabricated by dry nanomilling from different MNP systems.

MNP *β*-As_4_S_4_-Bearing Nanocomposite, Ref.	FSDP Parameters						
	2*Θ*,	FWHM,	*Q*, Å^−1^	Δ*Q*, Å^−1^	*R*, Å	*L*, Å	*d_s_*, Å
Bulk unmilled *β*-As_4_S_4_, [49,50]	16.712 (13)	4.35 (3)	1.185	0.31	5.30	20.3	6.52
Mono-particulate *β*-As_4_S_4_, [20,49]	16.213 (7)	3.57 (2)	1.150	0.25	5.46	24.7	6.72
Biparticulate 4·*β*-As_4_S_4_/1·Fe_3_O_4_, [20]	16.197 (11)	3.93 (4)	1.149	0.28	5.47	22.5	6.73
Biparticulate *β*-As_4_S_4_/Fe_3_O_4_, this study	16.068 (28)	4.64 (11)	1.140	0.33	5.51	19.0	6.78
Triparticulate *β*-As_4_S_4_/4·ZnS/Fe_3_O_4_, [23]	15.077 (3)	5.68 (14)	1.070	0.40	5.87	24.2	7.22

## Data Availability

The data presented in this study are available on request from the corresponding author. The data are not publicly available due to privacy or ethical reasons.
